# Primary growth hormone insensitivity (Laron syndrome) and acquired hypothyroidism: a case report

**DOI:** 10.1186/1752-1947-5-301

**Published:** 2011-07-11

**Authors:** Oana R Cotta, Libero Santarpia, Lorenzo Curtò, Gianluca Aimaretti, Ginevra Corneli, Francesco Trimarchi, Salvatore Cannavò

**Affiliations:** 1 Department of Medicine and Pharmacology, University of Messina, Messina, Italy; 2 Translational Research Unit, Department of Oncology Hospital of Prato and Istituto Toscano Tumori, Firenze, Italy; 3 Endocrinology, Department of Experimental and Clinical Medicine, University A. Avogadro del Piemonte Orientale, Novara, Italy; 4 Division of Endocrinology and Metabolism, Department of Internal Medicine, University of Turin, Turin, Italy

## Abstract

**Introduction:**

Primary growth hormone resistance or growth hormone insensitivity syndrome, also known as Laron syndrome, is a hereditary disease caused by deletions or different types of mutations in the growth hormone receptor gene or by post-receptor defects. This disorder is characterized by a clinical appearance of severe growth hormone deficiency with high levels of circulating growth hormone in contrast to low serum insulin-like growth factor 1 values.

**Case presentation:**

We report the case of a 15-year-old Caucasian girl who was diagnosed with Silver-Russell syndrome at the age of four and a half years. Recombinant growth hormone was administered for 18 months without an appropriate increase in growth velocity. At the age of seven years, her serum growth hormone levels were high, and an insulin-like growth factor 1 generation test did not increase insulin-like growth factor 1 levels (baseline insulin-like growth factor 1 levels, 52 μg/L; reference range, 75 μg/L to 365 μg/L; and peak, 76 μg/L and 50 μg/L after 12 and 84 hours, respectively, from baseline). The genetic analysis showed that the patient was homozygous for the R217X mutation in the growth hormone receptor gene, which is characteristic of Laron syndrome. On the basis of these results, the diagnosis of primary growth hormone insensitivity syndrome was made, and recombinant insulin-like growth factor 1 therapy was initiated. The patient's treatment was well tolerated, but unexplained central hypothyroidism occurred at the age of 12.9 years. At the age of 15 years, when the patient's sexual development was almost completed and her menstrual cycle occurred irregularly, her height was 129.8 cm, which is 4.71 standard deviations below the median for normal girls her age.

**Conclusion:**

The most important functional tests for the diagnosis of growth hormone insensitivity are the insulin-like growth factor 1 generation test and genetic analysis. Currently, the only effective treatment is daily administration of recombinant insulin-like growth factor 1 starting from early childhood. However, these patients show a dramatically impaired final height. In our case, unexplained central hypothyroidism occurred during treatment.

## Introduction

Primary growth hormone (GH) insensitivity (Laron syndrome) includes a range of disorders with demonstrable resistance to the action of GH. The classical GH insensitivity syndrome (GHIS) is an autosomal, recessively inherited form of dwarfism phenotypically resembling GH deficiency, but differing from it by high levels of circulating GH.

In 1966, the description of the first cases, three Yemenite Jewish siblings, led to the discovery of the polymorphic defects of the GH receptor (GHR) which result in the inability to generate insulin-like growth factor 1 (IGF-1) [1]. Nowadays, this disorder has been reported in more than 250 cases worldwide, being found mainly in consanguineous families from Mediterranean, Middle Eastern, or South Asian regions or in their descendants, including a large cohort identified in southern Ecuador who are considered to be descendants of *conversos *(Spanish Jews who became Catholic during the Inquisition) [2]. To date, more than 70 unique GHR mutations have been identified in more than 250 GHIS patients. These include missense or non-sense mutations, splice site mutations, and insertions or deletions [3,4], and the vast majority of the point mutations have compromised the extracellular domain. These mutations are almost all recessively inherited in either homozygous or compound heterozygous form.

We present the outcome of eight-year recombinant IGF-1 (rIGF-1) replacement in a girl with Laron type dwarfism caused by an R217X mutation of the gene encoding for GHR, who developed hypothyroidism during treatment.

## Case presentation

A 13-year-old Caucasian girl was referred to our unit for the follow-up of Laron-type dwarfism diagnosed six years earlier. She was the second child of a Sicilian family of second-degree cousins. Both parents' heights were in the normal range: her father was 178 cm tall, and her mother was 160 cm tall, and they were 37 and 31 years old, respectively, at the time of conception. As the product of a 36-week, uneventful gestation and delivery, the patient was born with normal birth weight and size. Failure to thrive became evident after the first year of life, when both her height (61 cm, -7.06 standard deviations (SDs)) and weight (6610 g) were well below the third percentile. Her head circumference (45 cm) was in the third percentile but appeared disproportionally large for her body. The frontal fontanel was open (1.5 cm × 1.5 cm), and pale skin, frontal bossing, blue sclera, a hypoplastic nasal bridge, obesity, and an increased upper-to-lower segment ratio were also noted. No hypoglycemic episode had been reported. Her routine blood analysis results were normal. Karyotype analysis revealed a normal, 46, XX female, and wrist radiography documented delayed bone maturation.

At the age of three and a half years, she underwent an endocrine evaluation in a pediatric center. Her baseline serum GH levels were 6 ng/mL and peaked at 8.5 ng/mL during a clonidine test (150 mg/m^2^, orally). Her serum IGF-1 values were very low (31 ng/mL and 82.9 ng/mL on two different days; reference range, 100 ng/mL to 500 ng/mL). Magnetic resonance imaging (MRI) showed a hypoplastic pituitary gland (images not available). At the age of four and a half years, she was diagnosed with Silver-Russell syndrome, and recombinant GH (rGH) was administered for 18 months without an appropriate increase in height velocity (3.5 cm/year, -3.20SD).

**Figure 1:**
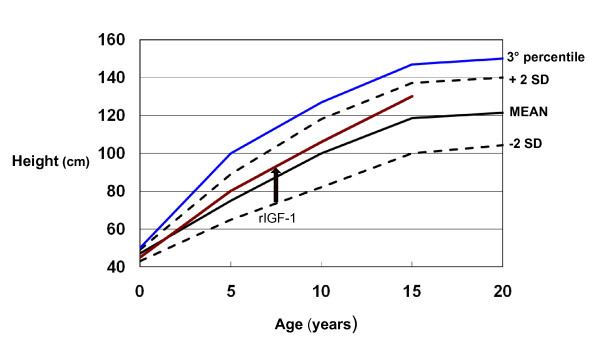
**Growth chart of our patient with primary growth hormone insensitivity syndrome (red line) before and during recombinant insulin-like growth factor 1 therapy**. (Reproduced with permission from Laron *et al*. [6].)

She was reevaluated at the age of seven years in an endocrine unit in another Italian city. Her serum GH level was high (26.8 ng/mL, representing the mean of three determinations during the same morning). An IGF-1 generation test (rGH, 0.03 mg/kg for four consecutive evenings) did not increase her IGF-1 levels (baseline IGF-1 level, 52 μg/L; reference range, 75 μg/L to 365 μg/L; peak, 76 μg/L and 50 μg/L after 12 and 84 hours, respectively, from the baseline). The genetic analysis showed that the patient was homozygous for the R217X mutation of the *GHR *gene [5], which is characteristic in patients with Laron syndrome. On the basis of these results, the diagnosis of primary GHIS was made and rIGF-1 therapy was initiated when she was seven and a half years of age. During the first year of treatment, her growth velocity showed a twofold increase. However, she did not experience appropriate catch-up growth, and at the age of 15 years, her height was 129.8 cm, 4.71SDs below the median for normal height (Figure 1). The starting dose of rIGF-1 was 50 μg/kg twice daily, and the dose was titrated according to GH and IGF-1 levels to a maximum dose of 120 μg/kg twice daily. Treatment was administered continuously until she was 13 and a half years of age, when her treatment was withdrawn because of drug unavailability for approximately six months. To date, our patient is still in therapy. She reported no adverse effects, no hypoglycemic episodes occurred, and she did not experience arthralgia, myalgia, or skeletal pain. Periodic evaluation excluded intra-cranial hypertension. An otorhinolaryngologic evaluation revealed the presence of tonsillar and adenoidal hypertrophy after approximately eight years of rIGF-1 therapy.

At the age of 12.9 years, when she was referred to us, an endocrinologic evaluation showed a low serum-free thyroxine (FT4) level (9.96 pmol/L; reference range, 12 pmol/L to 22 pmol/L). Her serum-free triiodothyronine (FT3) level (4.71 pmol/L; reference range, 3.0 pmol/L to 6.8 pmol/L) and thyroid-stimulating hormone (TSH) level (1.370 μIU/mL) were within the reference ranges. Previously, the patient's thyroid function had always been normal. Her test results for serum levels of thyroid peroxidase and thyroglobulin antibodies had always been negative. Thyroid ultrasonography showed a reduced thyroid size (right lobe transverse diameter (TD) 11 mm and anteroposterior diameter (APD) 13 mm; left lobe TD 10 mm and APD 9 mm) with normal echotexture and without thyroid nodules. She was prescribed levothyroxine (LT4) therapy (1.25 μg/kg/day), which resulted in normalization of her FT4 levels (12.07 pmol/L) and simultaneous suppression of TSH values (0.3 μIU/mL). Exogenous thyrotropin-releasing hormone administration (200 μg intravenously) induced a normal TSH response (peak at 20 minutes, 12.8 μIU/mL. An MRI study of her pituitary excluded any organic reason for central hypothyroidism. Puberty started six months later, and menarche occurred when she was 14.3 years of age. At the age of 15 years, the patient's sexual development was almost completed (Tanner stage 4), and her menstrual cycle occurred irregularly.

## Discussion

Our patient has a typical case of Laron syndrome presenting with a classical GHIS phenotype and laboratory findings, but unfortunately was diagnosed and therefore treated at a late age. Clinically, patients with GHIS present in a manner virtually indistinguishable from those with severe GH deficiency. Birth weight and length are likely to be within the reference ranges, but post-natal linear growth is strikingly abnormal with a rapid decline in growth velocity soon after birth. The natural history, without proper treatment, results in an extremely short adult stature ranging between 4 and 10 SDs below the median for normal height [6]. Relative obesity is present at birth and increases with age, with a relative excess of adipose tissue in the context of thin bones and diminished muscular mass. The upper-to-lower segment ratio is increased with regard to sex and chronologic age, denoting short limbs for trunk size. Congenital malformations, craniofacial abnormalities, and other physical features may be noted at birth. Facial bone growth is particularly retarded, and fontanel closure is delayed, leading to a disproportionate cephalofacial relationship because of the decreased vertical dimension of the face, with frontal bossing, a saddle nose, shallow orbits, and the setting sun sign of the eyes. Blue sclera may be noted, particularly in patients of Mediterranean or Middle Eastern origin. Hair growth is quite sparse in infancy and through early childhood. It is silky and forms temporal and frontal recessions. Tooth development is delayed, and the teeth may often be defective. The larynx is narrow, resulting in a very high-pitched voice. The genitalia and gonads are small from birth. Pubertal development is delayed, and the pubertal growth spurt is absent, but adult sexual maturation is eventually achieved. Walking and other gross motor developmental milestones are delayed because of the underdeveloped musculature. The hands and feet are small (acromicria). Hip dysplasia, notably avascular necrosis of the femoral head, has been reported in up to 25% of patients [2]. The skin is thin and has a fine texture with wrinkles as in premature aging. Cardiological investigations and pulmonary function studies have revealed cardiomicria, reduced width of the cardiac muscle, reduced left ventricular output, and reduced maximal aerobic capacity [7]. Psychological evaluations suggest a great variability in intellectual development, ranging from normal intelligence to severe mental retardation [3].

The cardinal biochemical features of GHIS are low levels of all GH-dependent proteins, including very low or even undetectable serum IGF-1 levels, IGF binding protein 3, and acid labile subunit in association with normal or increased GH levels. The regulation of GH secretion and feedback mechanisms is normal. The most important functional test for the diagnosis is the IGF-1 generation test because serum IGF-1 levels are low and do not increase with the administration of exogenous rGH for days or weeks, demonstrating the state of GH resistance in these patients [3]. Metabolic abnormalities include fasting hypoglycemia and hypercholesterolemia.

The underlying metabolic defect lies in the lack of responsiveness of the target organs to endogenous GH. In 1984, it was proven by liver biopsy that GH does not bind to its receptors and therefore is unable to generate IGF-1 [8]. This explains why patients with primary GHIS typically have low to undetectable serum levels of IGF-1, even though serum GH levels are normal or high. In addition, exogenous rGH fails to accelerate growth or to stimulate serum IGF-1 levels or IGF binding protein 3 [3]. At the age of four and a half years, our patient had been treated with rGH for a period of 18 months, but this therapy did not appropriately increase her growth velocity.

The only effective treatment is the daily administration of rIGF-1 starting from early childhood and probably throughout life. The rIGF-1 treatment accelerates linear growth velocity, and appropriate dose titrating results in tripling of the baseline growth rate during the first year of treatment [9]. Even if these patients may never experience sufficient catch-up growth to bring their height within the normal range, they do achieve an adult height significantly greater than expected in the absence of therapy [9]. The main reasons could be, on the one hand, the inability to replicate physiological IGF-1 distribution and action and, on the other hand, the inability to restore GH defects, because animal studies indicate that GH has growth-promoting effects apart from the IGFs [10].

Evidence exists that rIGF-1 therapy also reduces body fat, stimulates kidney function, and maintains left ventricle dimension and function within the normal range of age-matched control subjects [11].

Hypoglycemia is the most frequent side effect, observed both before and during therapy and reported in as many as 50% of cases. Lymphoid tissue hypertrophy associated with hypoacusis and snoring occurs in approximately 22% of treated patients, and tonsillar or adenoidal hypertrophy requiring tonsillectomy or adenoidectomy has been seen in 10% of cases. Several cases of intra-cranial hypertension or papilledema have been observed. Increased growth of the internal organs, which was a main concern before long-term trials were conducted, has been reported but with no clinical impact, because this effect waned over time. Acromegaloid coarsening of the face has also been reported in a number of patients, particularly those of pubertal age [9].

Our patient has been treated with rIGF-1 for approximately eight years, but she has not experienced enough catch-up growth to bring her height into the normal range. At the age of 15 years, she was 129.8 cm tall, which is 4.71 SDs below the median for normal height at her age. Except for tonsillar and adenoidal hypertrophy, no other known side effect has been reported.

The present knowledge of the effects of GH and IGF-1 deficiency on aging and lifespan suggests that untreated patients with congenital isolated IGF-1 deficiency seem to reach old age despite marked obesity, development of hyperlipidemia, and a tendency to develop diabetes and its complications, probably because the risk for cancer, a frequent cause of death in the general population, seems to be reduced in these patients [12].

Genetic analysis showed that our patient is homozygous for the R217X mutation in the *GHR *gene (homozygous C to T transition in exon 7 causing CGA to TGA substitution at codon 7) [5]. The molecular defect occurs in the extracellular domain of the GHR and leads to a premature termination signal and a truncated non-functional receptor.

This particular mutation has been reported previously in several patients from countries located in the Mediterranean and Middle Eastern region, as well as in North America [4,13]. The R217X mutation has been linked to the development of type 2 diabetes mellitus complicated by diabetic retinopathy in a patient with Laron-type dwarfism who had never been treated with rIGF-1 [14]. The patient had background diabetic retinopathy and progressively developed exudates, microaneurysms, hemorrhages, and clinically significant macular edema. He also had subacute ischemic heart disease. This suggests that congenital IGF-I deficiency, similar to excess, may cause vascular complications of diabetes mellitus, also denoting that vascular endothelial growth factor can induce neovascularization in the presence of congenital IGF-I deficiency [14].

At the age of 12.9 years, our patient suddenly developed hypothyroidism. Both low levels of FT4 associated with normal TSH levels present at diagnosis and TSH suppression after a relatively low LT4 dose per kilogram reinforced the hypothesis of central hypothyroidism.

It seems unlikely that her hypothyroidism was induced by rIGF-1 therapy. In support of this theory is the fact that during rIGF-1 treatment withdrawal for approximately six months, the patient's thyroid function did not improve. During that time, we also performed LT4 withdrawal, which did not result in an increase in FT4. Our finding is in agreement with that of Klinger *et al*. [15], who previously showed that rIGF-1 therapy does not cause abnormal thyroid function.

To the best of our knowledge, this is the first report of a case of GHIS associated with hypothyroidism. It remains to be seen whether there is any association with GHIS or whether it is an isolated case.

## Conclusions

Primary GH resistance or GHIS, also known as Laron syndrome, is a hereditary disease caused by deletions or mutations in the *GHR *gene or the post-receptor mechanisms. These polymorphic defects lead to the inability to generate IGF-1, which is the anabolic effector of GH. The early and continuous IGF-1 deficiency causes dwarfism as well as skeletal and muscular underdevelopment.

Daily administration of rIGF-1 is effective in promoting catch-up growth and is safe. However, no data are available concerning treatment throughout life. An early correct diagnosis of this syndrome is crucial for appropriate preventive care and therapy.

### Patient's perspective

**"**I am a 15-year-old girl and for the last 10 years of my life, twice daily, I am given an injection that is supposed to help me grow and make up for the height difference that distinguishes me from the other girls my age. I cannot consider myself too satisfied by this therapy, because I did not get the results I was hoping for, but on the other hand I realize that this drug is my only chance to gain height.

"I have always been taken care of by fine doctors. They helped me a lot and I take this chance to thank them all, both doctors from Turin as well as from Messina.

"As far as my daily life is concerned, I can say that, despite everything, I have no problems interacting with my friends. They appreciate me for who I am, because both from a psychological and intellectual point of view I honestly do not think I have any difficulties. My strength relies in my strong character and in the fact that, I am aware that there are many other children who have problems, probably more serious than mine.

"For the future I hope that research, based also on real life experience, can progress and help all children born with my same problem."

## Abbreviations

APD: anteroposterior diameter; FT3: free triiodothyronine; FT4: free thyroxine; GH: growth hormone; GHIS: growth hormone insensitivity syndrome; GHR: growth hormone receptor; IGF-1: insulin-like growth factor 1; LT4: levothyroxine; MRI: magnetic resonance imaging; rGH: recombinant growth hormone; rIGF-1: recombinant insulin-like growth factor 1; TD: transversal diameter; TSH: thyroid-stimulating hormone.

## Consent

Written informed consent was obtained from the patient's father for publication of this case report and any accompanying images.

## Competing interests

The authors declare that they have no competing interests.

## Authors' contributions

All authors contributed to the management of the patient. In addition, ORC collected data regarding the patient and wrote the manuscript. LS and LC reviewed the literature concerning the case. FT was a major contributor to the writing of the manuscript. SC analyzed the data and supervised the editing of the manuscript. All authors read and approved the final manuscript.

## References

[B1] Laron Z Pertzelan A Mannheimer S Genetic pituitary dwarfism with high serum concentration of growth hormone: a new inborn error of metabolism? Isr J Med Sci 1966 2 152 155 5916640

[B2] Rosenbloom AL Guevara-Aguirre J Rosenfeld RG Francke U Growth hormone receptor deficiency in Ecuador J Clin Endocrinol Metab 1999 84 4436 4443 10.1210/jc.84.12.4436 10599699

[B3] Laron Z Laron syndrome (primary growth hormone resistance or insensitivity): the personal experience 1958-2003 J Clin Endocrinol Metab 2004 89 1031 1044 10.1210/jc.2003-031033 15001582

[B4] Savage MO Attie KM David A Metherell LA Clark AJ Camacho-Hübner C Endocrine assessment, molecular characterization and treatment of growth hormone insensitivity disorders Nat Clin Pract Endocrinol Metab 2006 2 395 407 10.1038/ncpendmet0195 16932322

[B5] Fassone L Corneli G Bellone S Camacho-Hübner C Aimaretti G Cappa M Ubertini G Bona G Growth hormone receptor gene mutations in two Italian patients with Laron syndrome J Endocrinol Invest 2007 30 417 420 17598975 10.1007/BF03346320

[B6] Laron Z Lilos P Klinger B Growth curves for Laron syndrome Arch Dis Child 1993 68 768 770 10.1136/adc.68.6.768 8333769 PMC1029371

[B7] Ben-Dov I Gaides M Scheinowitz M Wagner R Laron Z Reduced exercise capacity in untreated adults with primary growth hormone resistance (Laron syndrome) Clin Endocrinol (Oxf) 2003 59 763 767 10.1046/j.1365-2265.2003.01920.x 14974919

[B8] Eshet R Laron Z Pertzelan A Dintzman M Defect of human growth hormone in the liver of two patients with Laron type dwarfism Isr J Med Sci 1984 20 8 11 6321400

[B9] Chernausek SD Backeljauw PF Frane J Kuntze J Underwood LE GH Insensitivity Syndrome Collaborative Group Long-term treatment with recombinant insulin-like growth factor (IGF)-I in children with severe IGF-I deficiency due to growth hormone insensitivity J Clin Endocrinol Metab 2007 3 902 910 10.1210/jc.2006-1610 17192294

[B10] Lupu F Terwilliger JD Lee K Segre GV Efstratiadis A Roles of growth hormone and insulin-like growth factor 1 in mouse postnatal growth Dev Biol 2001 229 141 162 10.1006/dbio.2000.9975 11133160

[B11] Scheinowitz M Feinberg MS Laron Z IGF-I replacement therapy in children with congenital IGF-I deficiency (Laron syndrome) maintains heart dimension and function Growth Horm IGF Res 2009 19 280 282 10.1016/j.ghir.2008.11.004 19117781

[B12] Laron Z The GH-IGF1 axis and longevity: the paradigm of IGF1 deficiency Hormones 2008 7 24 27 18359741 10.14310/horm.2002.1111034

[B13] Berg MA Argente J Chernausek S Gracia R Guevara-Aguirre J Hopp M Pérez-Jurado L Rosenbloom A Toledo SP Francke U Diverse growth hormone receptor gene mutations in Laron syndrome Am J Hum Genet 1993 52 998 1005 8488849 PMC1682057

[B14] Laron Z Weinberger D Diabetic retinopathy in two patients with congenital IGF-I deficiency (Laron syndrome) Eur J Endocrinol 2004 151 103 106 10.1530/eje.0.1510103 15248828

[B15] Klinger B Ionesco A Anin S Laron Z Effect of insulin-like growth factor I on the thyroid axis in patients with Laron-type dwarfism and healthy subjects Acta Endocrinol (Copenh) 1992 127 515 519 10.1530/acta.0.1270515 1362849

